# How to Interpret the Head-Up Tilt Table Test: Teaching the 3 Core Orthostatic Hemodynamic Patterns

**DOI:** 10.1212/NE9.0000000000200315

**Published:** 2026-04-22

**Authors:** Priyanka Shekhawat, Aditi Varma-Doyle, Khosro Farhad, Nathaniel M. Robbins, Peter Novak

**Affiliations:** 1 Mass General Brigham Wentworth Douglass Hospital;; 2 Department of Neurology, Mass General Brigham, Boston, MA; and; 3 Harvard Medical School, Boston, MA.

**Figure 1:**
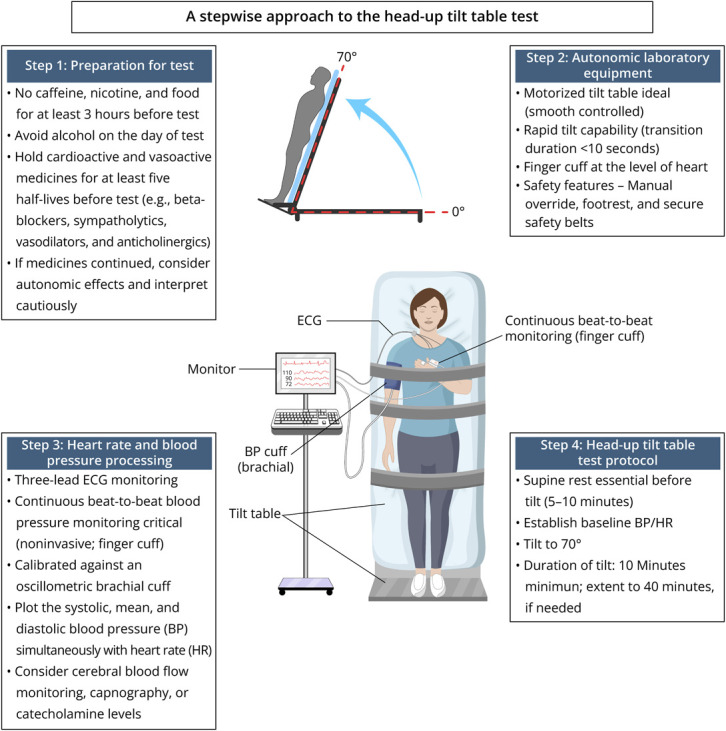
A Stepwise Approach to Head-Up Tilt Table Test Overview of patient preparation, autonomic laboratory setup, continuous heart rate and beat-to-beat blood pressure acquisition, and the standardized tilt protocol, providing a structured framework for accurate hemodynamic recording and interpretation of orthostatic responses.

The head-up tilt table test (HUTT) evaluates orthostatic intolerance.^1^ This Neurovisual presents a structured approach to patient preparation, tilt table setup, signal acquisition, and testing protocol (Figure 1); standardization of these elements is essential for accurate measurement and interpretation of orthostatic hemodynamic responses.

**Figure 2:**
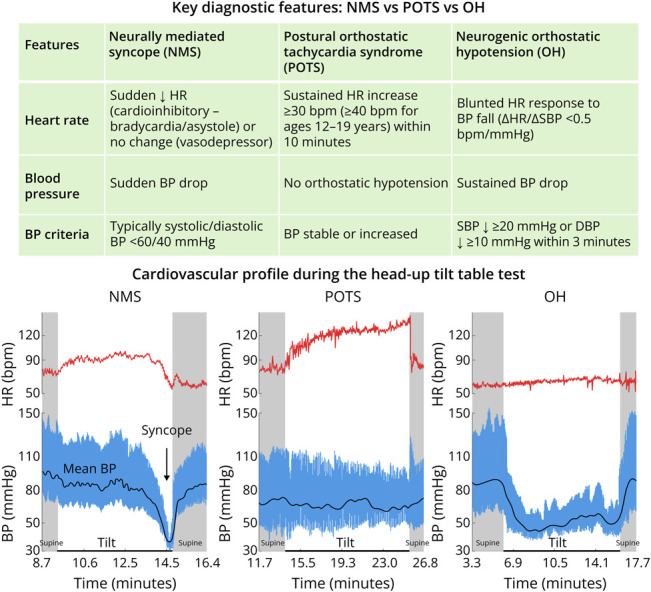
Interpreting Head-Up Tilt Table Test: Key Diagnostic Features Characteristic heart rate and beat-to-beat blood pressure responses in neurally mediated syncope, postural orthostatic tachycardia syndrome, and neurogenic orthostatic hypotension across supine, tilt, and recovery phases, with corresponding diagnostic criteria.

It then illustrates the principal patterns—neurally mediated syncope, postural orthostatic tachycardia syndrome, and neurogenic orthostatic hypotension—on continuous heart rate and beat-to-beat blood pressure recordings to facilitate pattern recognition (Figure 2).^2^ The temporal relationship between symptoms and hemodynamic changes is critical; symptom reproduction without corresponding physiologic abnormalities may suggest alternative diagnoses, whereas concordant changes support an autonomic etiology.
